# Supplementary material for: Resolving the full spectrum of human genome variation using Linked-Reads
Source: Genome Res. 2019 Apr;29(4):635–45. doi: 10.1101/gr.234443.118 (PMC6442396; doi:10.1101/gr.234443.118)
Supplement: Supplemental Material [file supp_gr.234443.118_Supplemental_longranger-2.2.zip › longranger-2.2.tar.bz2!/lr2.2-public/lib/stan/cmdstan/stan/doc/api/html/structstan_1_1math_1_1coupled__ode__system_3_01_f_00_01double_00_01double_01_4.html]

Stan: stan::math::coupled\_ode\_system< F, double, double > Struct Template Reference


|  |  |
| --- | --- |
|  | Stan  2.5.0  probability, sampling & optimization |


- Main Page
- Related Pages
- Namespaces
- Classes
- Files

- Class List
- Class Index
- Class Hierarchy
- Class Members

All Classes Namespaces Files Functions Variables Typedefs Enumerations Enumerator Friends Macros Pages

- stan
- math
- coupled\_ode\_system< F, double, double >

Public Member Functions |
Public Attributes |
List of all members

stan::math::coupled\_ode\_system< F, double, double > Struct Template Reference

The coupled ode system for known initial values and known parameters.
More...

`#include <coupled_ode_system.hpp>`

|  |  |
| --- | --- |
| Public Member Functions | |
|  | coupled\_ode\_system (const F &f, const std::vector< double > &y0, const std::vector< double > &theta, const std::vector< double > &x, const std::vector< int > &x\_int, std::ostream \*msgs) |
|  | Construct the coupled ODE system from the base system function, initial state, parameters, data and a stream for messages. More... |
|  | |
| void | operator() (const std::vector< double > &y, std::vector< double > &dy\_dt, double t) |
|  | Calculates the derivative of the coupled ode system with respect to the specified state at the specified time using the system state function. More... |
|  | |
| int | size () const |
|  | Returns the size of the coupled system. More... |
|  | |
| std::vector< double > | initial\_state () |
|  | Returns the initial state of the coupled system, which is identical to the base ODE original state in this implementation because the initial state is known. More... |
|  | |
| std::vector< std::vector  < double > > | decouple\_states (const std::vector< std::vector< double > > &y) |
|  | Returns the base portion of the coupled state. More... |
|  | |

|  |  |
| --- | --- |
| Public Attributes | |
| const F & | f\_ |
|  | |
| const std::vector< double > & | y0\_dbl\_ |
|  | |
| const std::vector< double > & | theta\_dbl\_ |
|  | |
| const std::vector< double > & | x\_ |
|  | |
| const std::vector< int > & | x\_int\_ |
|  | |
| const int | N\_ |
|  | |
| const int | M\_ |
|  | |
| const int | size\_ |
|  | |
| std::ostream \* | msgs\_ |
|  | |

## Detailed Description

### template<typename F> struct stan::math::coupled\_ode\_system< F, double, double >

The coupled ode system for known initial values and known parameters.

This coupled system does not add anything to the base system used to construct it, but is here for generality of the integration implementation.

Template Parameters
:   |  |  |
    | --- | --- |
    | F | type of system function for the base ODE system. |

Definition at line 42 of file coupled\_ode\_system.hpp.

## Constructor & Destructor Documentation

template<typename F >

|  |  |  |  |
| --- | --- | --- | --- |
| stan::math::coupled\_ode\_system< F, double, double >::coupled\_ode\_system | ( | const F & | *f*, |
|  |  | const std::vector< double > & | *y0*, |
|  |  | const std::vector< double > & | *theta*, |
|  |  | const std::vector< double > & | *x*, |
|  |  | const std::vector< int > & | *x\_int*, |
|  |  | std::ostream \* | *msgs* |
|  | ) |  |  |

inline

Construct the coupled ODE system from the base system function, initial state, parameters, data and a stream for messages.

Parameters
:   |  |  |  |
    | --- | --- | --- |
    | [in] | f | base ode system functor. |
    | [in] | y0 | initial state of the base ode. |
    | [in] | theta | parameters of the base ode. |
    | [in] | x | real data. |
    | [in] | x\_int | integer data. |
    | [in,out] | msgs | print stream. |

Definition at line 66 of file coupled\_ode\_system.hpp.

## Member Function Documentation

template<typename F >

|  |  |  |  |  |  |
| --- | --- | --- | --- | --- | --- |
| std::vector<std::vector<double> > stan::math::coupled\_ode\_system< F, double, double >::decouple\_states | ( | const std::vector< std::vector< double > > & | *y* | ) |  |

inline

Returns the base portion of the coupled state.

In this class's implementation, the coupled system is equivalent to the base system, so this function just returns its input.

Parameters
:   |  |  |
    | --- | --- |
    | y | the vector of the coupled states after solving the ode |

Returns
:   the decoupled states

Definition at line 146 of file coupled\_ode\_system.hpp.

template<typename F >

|  |  |  |  |  |
| --- | --- | --- | --- | --- |
| std::vector<double> stan::math::coupled\_ode\_system< F, double, double >::initial\_state | ( |  | ) |  |

inline

Returns the initial state of the coupled system, which is identical to the base ODE original state in this implementation because the initial state is known.

The return value is a vector of length size() where the first N (base ode system size) parameters are the initial conditions of the base ode system and the rest of the initial conditions is 0.

Returns
:   initial state of the coupled system

Definition at line 128 of file coupled\_ode\_system.hpp.

template<typename F >

|  |  |  |  |
| --- | --- | --- | --- |
| void stan::math::coupled\_ode\_system< F, double, double >::operator() | ( | const std::vector< double > & | *y*, |
|  |  | std::vector< double > & | *dy\_dt*, |
|  |  | double | *t* |
|  | ) |  |  |

inline

Calculates the derivative of the coupled ode system with respect to the specified state at the specified time using the system state function.

The derivative vector created is the same length as the length as the state vector.

Parameters
:   |  |  |  |
    | --- | --- | --- |
    | [in] | y | current state of the coupled ode. |
    | [out] | dy\_dt | populated with derivatives of the coupled system evaluated at specified state and time. |
    | [in] | t | time. |

Exceptions
:   |  |  |
    | --- | --- |
    | exception | if the system function does not return a derivative vector of the same size as the state vector. |

Definition at line 98 of file coupled\_ode\_system.hpp.

template<typename F >

|  |  |  |  |  |
| --- | --- | --- | --- | --- |
| int stan::math::coupled\_ode\_system< F, double, double >::size | ( |  | ) | const |

inline

Returns the size of the coupled system.

Returns
:   size of the coupled system.

Definition at line 112 of file coupled\_ode\_system.hpp.

## Member Data Documentation

template<typename F >

const F& stan::math::coupled\_ode\_system< F, double, double >::f\_

Definition at line 44 of file coupled\_ode\_system.hpp.

template<typename F >

const int stan::math::coupled\_ode\_system< F, double, double >::M\_

Definition at line 50 of file coupled\_ode\_system.hpp.

template<typename F >

std::ostream\* stan::math::coupled\_ode\_system< F, double, double >::msgs\_

Definition at line 52 of file coupled\_ode\_system.hpp.

template<typename F >

const int stan::math::coupled\_ode\_system< F, double, double >::N\_

Definition at line 49 of file coupled\_ode\_system.hpp.

template<typename F >

const int stan::math::coupled\_ode\_system< F, double, double >::size\_

Definition at line 51 of file coupled\_ode\_system.hpp.

template<typename F >

const std::vector<double>& stan::math::coupled\_ode\_system< F, double, double >::theta\_dbl\_

Definition at line 46 of file coupled\_ode\_system.hpp.

template<typename F >

const std::vector<double>& stan::math::coupled\_ode\_system< F, double, double >::x\_

Definition at line 47 of file coupled\_ode\_system.hpp.

template<typename F >

const std::vector<int>& stan::math::coupled\_ode\_system< F, double, double >::x\_int\_

Definition at line 48 of file coupled\_ode\_system.hpp.

template<typename F >

const std::vector<double>& stan::math::coupled\_ode\_system< F, double, double >::y0\_dbl\_

Definition at line 45 of file coupled\_ode\_system.hpp.

---

The documentation for this struct was generated from the following file:

- src/stan/math/ode/coupled\_ode\_system.hpp


---

[ Stan Home Page ]

*© 2011–2014,
Stan Development Team.*
